# Population genetic structure and phylogenetic analysis of *Anopheles hyrcanus* (Diptera: Culicidae) inferred from DNA sequences of nuclear ITS2 and the mitochondrial COI gene in the northern part of Iran

**DOI:** 10.1186/s12879-024-09626-0

**Published:** 2024-07-23

**Authors:** Fatemeh Askari, Azim Paksa, Saeed Shahabi, Shahin Saeedi, Aioub Sofizadeh, Mozaffar Vahedi, Aboozar Soltani

**Affiliations:** 1 Student Research Committee, Department of Biology and Control of Disease Vectors, School of Health, Shiraz University of Medical Sciences, Shiraz, Iran; 2 Department of Biology and Control of Disease Vectors, School of Health, Shiraz University of Medical Sciences, Shiraz, Iran; 3 Infectious Diseases Research Center, Golestan University of Medical Sciences, Gorgan, Iran; 4 Research Center for Health Sciences, Institute of Health, Department of Medical Entomology and Vector Control, School of Health, Shiraz University of Medical Sciences, Shiraz, Iran

**Keywords:** DNA barcoding, *Anopheles Hyrcanus*, ITS2, COI, Phylogenetic Analysis, Iran

## Abstract

**Background:**

The *Anopheles hyrcanus* group is distributed throughout the Oriental and Palaearctic regions and can transmit diseases such as malaria, Japanese encephalitis virus, and filariasis. This investigation marks the inaugural comprehensive study to undertake a phylogenetic analysis of the constituents of this malaria vector group in the northeastern region of Iran, juxtaposed with documented occurrences from different areas within Iran and worldwide.

**Methods:**

Mosquitoes were collected using various methods from nine different locations in Golestan province from April to December 2023. The collected mosquitoes were identified morphologically using valid taxonomic keys. DNA was isolated using the Sambio™ Kit. COI and ITS2 primers were designed using Oligo7 and GeneRunner. PCR and purification were performed with the Qiagen kit. Subsequently, sequencing was carried out at the Mehr Mam GENE Center using an Applied Biosystems 3730XL sequencer. The nucleotide sequences were then analyzed and aligned with GenBank data using BioEdit. Kimura 2-parameter was Utilized for base substitutions. DNA models were selected based on AIC and BIC criteria. Bayesian and Maximum Likelihood trees were constructed, along with a haplotype network. Molecular diversity statistics computed using DnaSP software.

**Results:**

In this study, a total of 819 adult mosquitoes were collected. *An. hyrcanus* was the second most abundant species, predominantly found in Kalaleh and Turkman counties. The sequenced and edited COI and ITS2 sequences were deposited in GenBank under specific accession numbers. Phylogenetic analyses using ML, BI, and NJ methods confirmed a monophyletic lineage for *An. hyrcanus* with strong support. Molecular analysis of Iranian *An. hyrcanus* found 11 diverse haplotypes, with the COI gene displaying low diversity. The ITS2 gene revealed two clades - one associating with Iran, Europe, and Asia; the other originating from southwestern Iran. The haplotype network showed two main groups - one from southwest Iran and the other from north Iran. Iran exhibited six distinct haplotypes, while Turkey showcased the highest diversity.

**Conclusions:**

*An. hyrcanus* in southwestern Iran exhibits a distinct haplogroup, suggesting possible subspecies differentiation. Additional studies are required to validate this phenomenon.

## Introduction

Mosquitoes are responsible for spreading many harmful pathogens and parasites, such as viruses, bacteria, protozoa, and nematodes, leading to diseases like malaria, dengue, yellow fever, encephalitis, and filariasis. Out of 3578 mosquito species, 88 are known to transmit 78 different pathogens that cause diseases in humans [1]. There are more than 3,600 species of mosquitoes categorized into 112 genera globally. The Anopheles, Culex, along with the Aedes, are significant genera of mosquito worldwide [2].

The Anopheles genus consists of a significant number of species found worldwide. Previous research has also shown the varied abundance and distribution of Anopheles species in different regions of the world. Furthermore, a study on the viruses carried by Anopheles mosquitoes identified 161 viruses in 54 Anopheles species from 41 countries across the globe, highlighting the diverse nature of Anopheles species worldwide. The Anopheles genus is known to include about 500 officially named species and 49 subspecies, with various subgenera containing named species of medical or veterinary significance [3–5].

In southwestern Asia, there are seven genera and over 98 species of mosquitoes belonging to the Culicidae family. The most recent checklist of Iranian mosquitoes includes species from both subfamilies, totaling 70 species representing 8 or 12 genera, depending on the classification of aedines [6, 7]. In the southeast of Iran, *Anopheles fluviatilis*, *An. dthali*, *An. stephensi*, *An. culicifacies*, and *An. superpictus* are recognized as main malaria vectors. While *Anopheles hyrcanus* species is not known as the main malaria vector in Iran, a very similar vector species has been reported for Afghanistan, with the only difference being *An. hyrcanus* instead of *An. dthali* as the vector in Afghanistan. [8].

Limited information is available regarding the mosquito fauna and phylogenetics in Golestan Province. So far, 10 *Anopheles* and 14 Culicinae species have been identified based on their morphological characteristics and the surface patterns of their eggs. Previous research has focused on other northeastern parts of Iran, including Mazandaran and North Khorasan Provinces [9].

The Hyrcanus group contains cryptic species that cannot be differentiated based on morphological characteristics and can only be identified through gene sequencing [10–12]. Additionally, there is ongoing debate about the evolutionary relationships between certain species within the group. To address this, researchers have utilized mitochondrial gene fragment sequences and internally transcribed spacer (ITS) sequences for phylogenetic analysis of the Hyrcanus group [12, 13].

*An. hyrcanus* and *An. pseudopictus* were previously considered as distinct species, but findings from Ponçon et al. suggest that the genetic differences in the internal transcribed spacer 2 (ITS2), cytochrome c oxidase I (COI), and cytochrome oxidase subunit II (COII) sequences between the two species fall within the range of differences typically seen within a single species [14]. Following this, Djadid et al. compared the ITS2 sequences of *An. hyrcanus* and *An. pseudopictus* in Iran, and their results also supported the idea that these two are the same species [15].

Studying mosquitoes in a systematic way is crucial for understanding their biology and effectively managing diseases they transmit. The taxonomy of Culicidae has been a focus of attention due to their role in spreading diseases, making it an important area of research for medical and veterinary purposes [16]. Investigating the diversity of species allows for studying their distribution in the environment and providing recommendations for controlling medically significant species.

Due to the significance of the Hyrcanian forests in northern Iran and the importance of species diversity within this area, a comprehensive study of the Hyrcanus group and its phylogeny in Golestan province (northeast Iran) had not been conducted until now. Therefore, this study represents the first comprehensive research to perform a phylogenetic analysis of the members of this malaria vector group in the northeast of Iran and to compare it with recorded samples from various regions in Iran and globally.

## Materials and methods

### Study area

Golestan province, situated in the northeast of Iran along the Caspian Sea, covers an area of approximately 20,437.74 km2 and comprises 11 districts with a population of around 1.6 million. The region’s diverse topography gives rise to three distinct climates: plain moderate, mountainous, and semi-arid, with an average annual rainfall of 556 mm and a temperature of 18.2 °C. The province is characterized by various land uses, including agricultural, industrial, urban, forest, range, and uncultivated lands. Major crops grown in Golestan include wheat, barley, cotton, soya beans, rice, and citrus fruits, while mining activities, such as coal and ballast mining, are concentrated in the central south and northeast of the province.

**Fig. 1:**
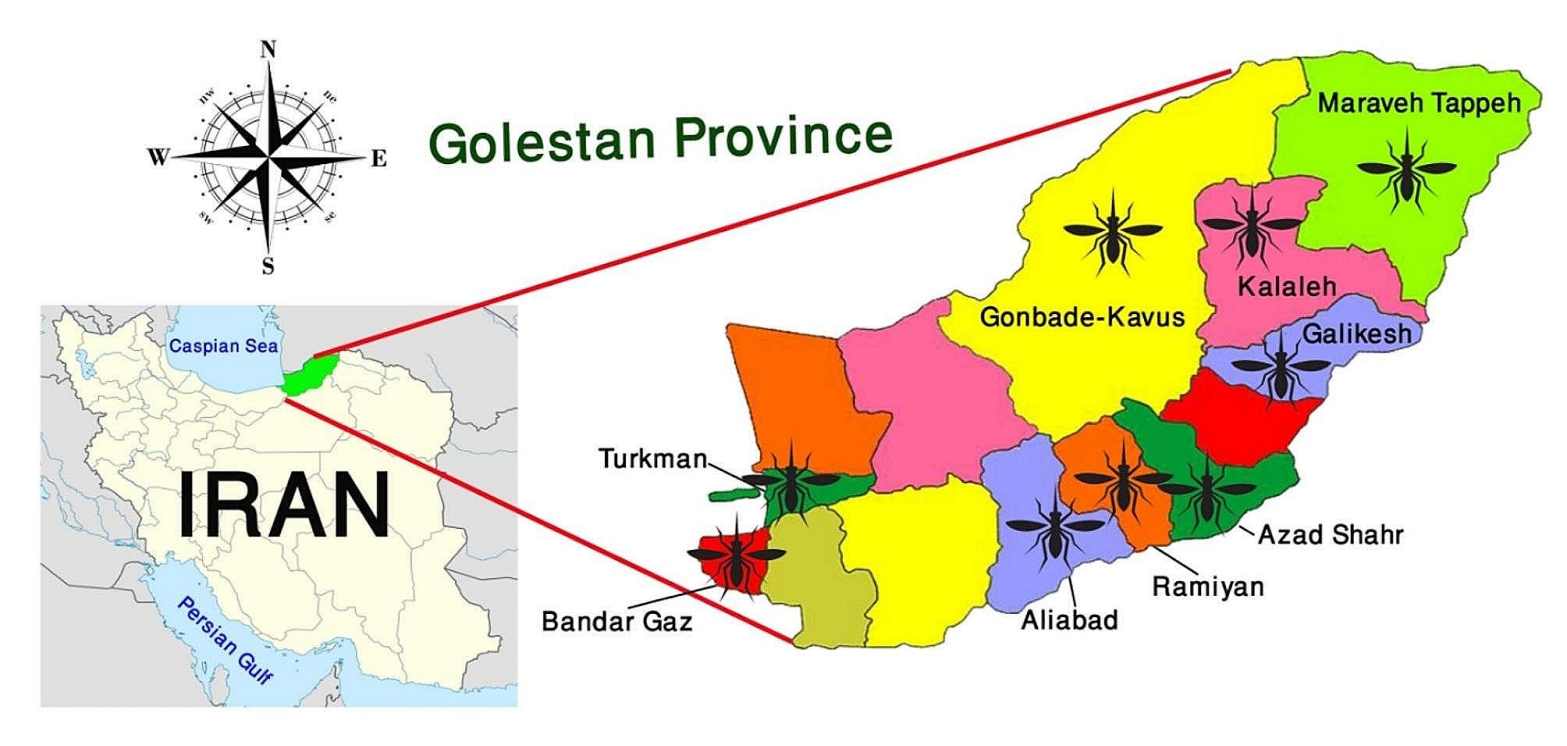
Map of Iran, highlighting the position of Golestan Province, northeasthern Iran, and its nine selected counties for sampling during, April–December 2023

Data was collected from nine locations in Golestan province from April to December 2023 (Fig. 1). To capture the diverse climatic conditions with specific ecological impacts for entomological studies, one or two cities from each of the main climates were chosen, with three locations sampled from each city. Thus, sampling was conducted in the southern regions (Ramiyan, and Aliabad), the northern region (Gonbade-Kavus), the western areas (Kordkuy, Bandar Gaz, and Turkmen), and the eastern locales (Galikesh, Maraveh Tappeh, and Kalaleh). The altitude, longitude, latitude and topography of the stations are listed in Table 1.

**Table 1:** Collection data for mosquitoes catches at sites in Golestan Province, northeastern Iran, April–December 2023

Locality (County)	Topography of sampling location	Coordinates	Altitude (m)
*Ramiyan*	Mountainous	37.01468500°N, 55.14038900°E	226
*Kordkuy*	Foothill	36.7917° N, 54.1133° E	50
*Aliabad*	Foothill	36.941857° N, 54.571879° E	140
*Gonbade-Kavus*	Plain	37.15° N, 55°E	52
*Bandar Gaz*	Coastal	36.7775° N, 53.9489° E	-14
*Turkman*	Coastal	36.909722° N, 54.113611° E	-20
*Galikesh*	Mountainous	37.2661° N, 55.4367° E	210
*Maraveh Tappeh*	Mountainous	37.90430400° N, 55.95454000° E	206
*Kalaleh*	Foothill	37.38207145° N, 55.49228362° E	160

### Sampling methods and species identification

Adult mosquitoes were captured using various methods, including hand catch, night and day-landing catch, total catch (pyrethrum spray sheet collection), UV light traps, and pit shelter trap collections. Hand catch collections involved the use of manual aspirators outdoors and indoors for an average of 20 min. UV light traps were employed every two weeks, and landing catches were conducted using human and animal baits. Additionally, pit shelters were dug in shady areas, and resting mosquitoes were collected using aspirators early in the morning. The collected mosquitoes were then transferred to the entomology laboratory for identification, and detailed geographic, climatic, and environmental characteristics of the sampling sites were recorded [6]. The collected mosquitoes were identified morphologically using valid taxonomic keys outlined in the works of Harbach from 1985 and Azari-Hamidian and Harbach from 2009 [17].

### DNA extraction

Genomic DNA was extracted from the entire unfed female mosquito using the Sambio™ Kit DNA extraction method according to the manufacturer’s instructions. Subsequently, the extracted DNA was subjected to 1% agarose gel electrophoresis to confirm its presence.

### COI and ITS2 PCR amplification and sequencing

The ITS2 and COI genes are commonly used markers for identifying mosquito species; all PCR products were conducted using a Flex Cycler2 thermal cycler. The PCR protocol for the COI gene involved an initial denaturation step at 94 °C for 3 min, followed by 40 cycles of 94 °C for 20 s, 55 °C for 20 s, 72 °C for 30 s, and a final extension at 72 °C for 5 min. The PCR protocol for the ITS2 gene included an initial denaturation step at 94 °C for 5 min, followed by 30 cycles of 94 °C for 30 s, 55 °C for 1 min, 72 °C for 30 s, and a final extension at 72 °C for 10 min. COI and ITS2 primers were designed using Oligo7 and GeneRunner softwares (Table 2).

**Table 2:** Primer list used in this study for PCRs and sequencing of COI and ITS2 gene

*n*	Gene	Primer	Sequence (5’-3’)
**1**	COI	COI F	TGG TCC AAT AAG TGA AGA AAC
		COI R	AAA AAT TAA ACG CTA CTC CA
**2**	ITS2	ITS2F	TTTAACATGCGCGCAAAAGG
		ITS2R	TAAGCCCAACAGTGAACATATG

PCR products were verified using 1% agarose gels containing DNA gel stain (Pishgam Company, Iran) and visualized under a UV light source (Analytik Jena, LLC, Jena, Germany). Samples showing visible bands were purified using a Qiagen QIAquick PCR purification kit following the manufacturer’s instructions (Qiagen Inc., Hilden, Germany). Following purification, the COI and ITS2 PCR products were sequenced at the Mehr Mam GENE Center DNA Sequencing Facility on an Applied Biosystems 3730XL DNA sequencer (Applied Biosystems, Foster City, CA, USA) using the same primers as for amplification. Both COI and ITS2 primers were utilized in this investigation, and subsequently, the sequences were deposited in the World GenBank. Extracted DNA of *Aedes caspius* and distilled water were used as positive and negative controls for PCR amplification, respectively.

### Phylogenetic and genetic analysis

The raw nucleotide sequences were examined, analyzed, and multiple-aligned with sequences retrieved from GenBank using BioEdit version 7.2.5 software. A Neighbor-Joining tree was reconstructed with the MEGAX software [18]. The number of base substitutions per site from averaging over all sequence pairs between species (Kimura 2-parameter genetic distance) were computed using the Kimura 2-parameter model. The best DNA substitution model was identified using both the Akaike (AIC) and Bayesian (BIC) information criteria using jModelTest, version 0.1.1 [19]. The best selected AIC model for COI and ITS2 sequences was TIM2 + I + G (-lnL = 2657.3634, K = 102, freqA = 0.3011, freqC = 0.143, freqG = 0.1596, freqT = 0.3964, *p*-inv = 0.5850, gamma shape = 0.7420), and HKY + G (-lnL = 1479.2558, K = 81, freqA = 0.297, freqC = 0.203, freqG = 0.2435, freqT = 0.2565, kappa = 1.5022 (ti/tv = 0.7524), gamma shape = 1.3150) respectively. The model of TPM2uf + I + G (-lnL = 2658.7821, K = 101, freqA = 0.2997, freqC = 0.1500, freqG = 0.1474, freqT = 0.4029, *p*-inv = 0.543, gamma shape = 0.563) with the lowest Bayesian information criterion (BIC) was selected for COI sequences. Bayesian inference (BI) with four search chains within each run for 10,000,000 generations, and Maximum Likelihood (ML) tree with 1000 replication, were reconstructed using the software MrBayes version 3.2, and PhyML version 3 [20] respectively. A haplotype network of ITS2 gene sequences was constructed using the median-joining approach available in the software PopART (Population Analysis with Reticulate Trees) [21]. Summary statistics of molecular diversity within 128 ITS2 sequences of *An. hyrcanus* from this study and all individuals from GenBank including total number of sites, number of haplotypes, polymorphic (segregating) sites, average number of nucleotide differences, nucleotide and haplotype diversity as well as the number of parsimony informative sites were measured using the software DnaSP version 6.0 [22].

## Results

A total of 819 adult mosquitoes were collected from all sampling locations in the current study. In our research, the pyrethrum spray sheet collection method displayed the highest capture rate (53% of all mosquitoes collected). Out of the total samples collected, 491 female mosquitoes were morphologically identified using valid taxonomic keys. Among the identified mosquitoes, *An. hyrcanus* was the second most abundant species (subdominant species) with 42 (8.55%) specimens collected. This species was found exclusively in Kalaleh (90.5%) and Turkman (9.5%) counties (Fig. 1). 20% of all collected specimens from various regions within the province were chosen for the molecular study. Following DNA extraction and PCRs, these specimens underwent sequencing analyses. Among them, 7 and 6 DNA sequences of COI and ITS2 gene, respectively, demonstrated high-quality of readings, which were used in the final molecular analyses.

### Phylogenetic relationship and genetic structure

The BLAST analysis, using both COI and ITS2 genes, confirmed the precise identification of these samples as *An. hyrcanus*, showing 98.4-99.64% and 98–100% similarity to GenBank sequences of COI and ITS2 genes, respectively. The PCR product underwent sequencing and editing, after which the edited COI and ITS2 sequences were submitted to the GenBank with accession numbers PP422139-45 and PP419983-88, respectively.

**Fig. 2:**
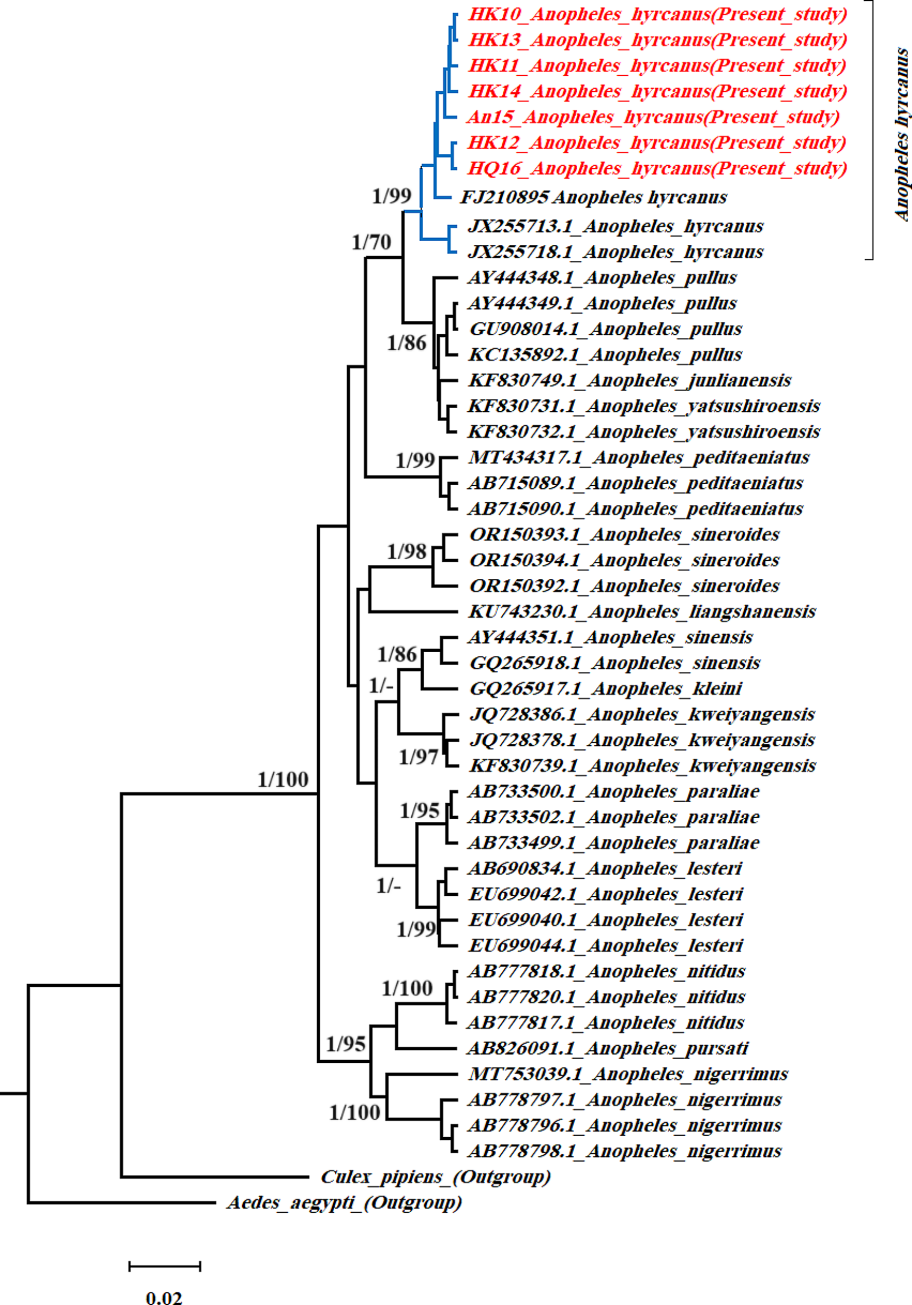
Bayesian phylogenetic relationship of the *Anopheles* species from Iran (present study accession numbers are shown in red) and other countries based on the *COI* gene sequences. Nodal support presented at the node indicates Bayesian posterior probability and bootstrap support for maximum likelihood inherence (1000 replicates). Values below 70% are not shown. *Culex pipiens* and *Aedes aegypti* were used as the out-group

**Fig. 3:**
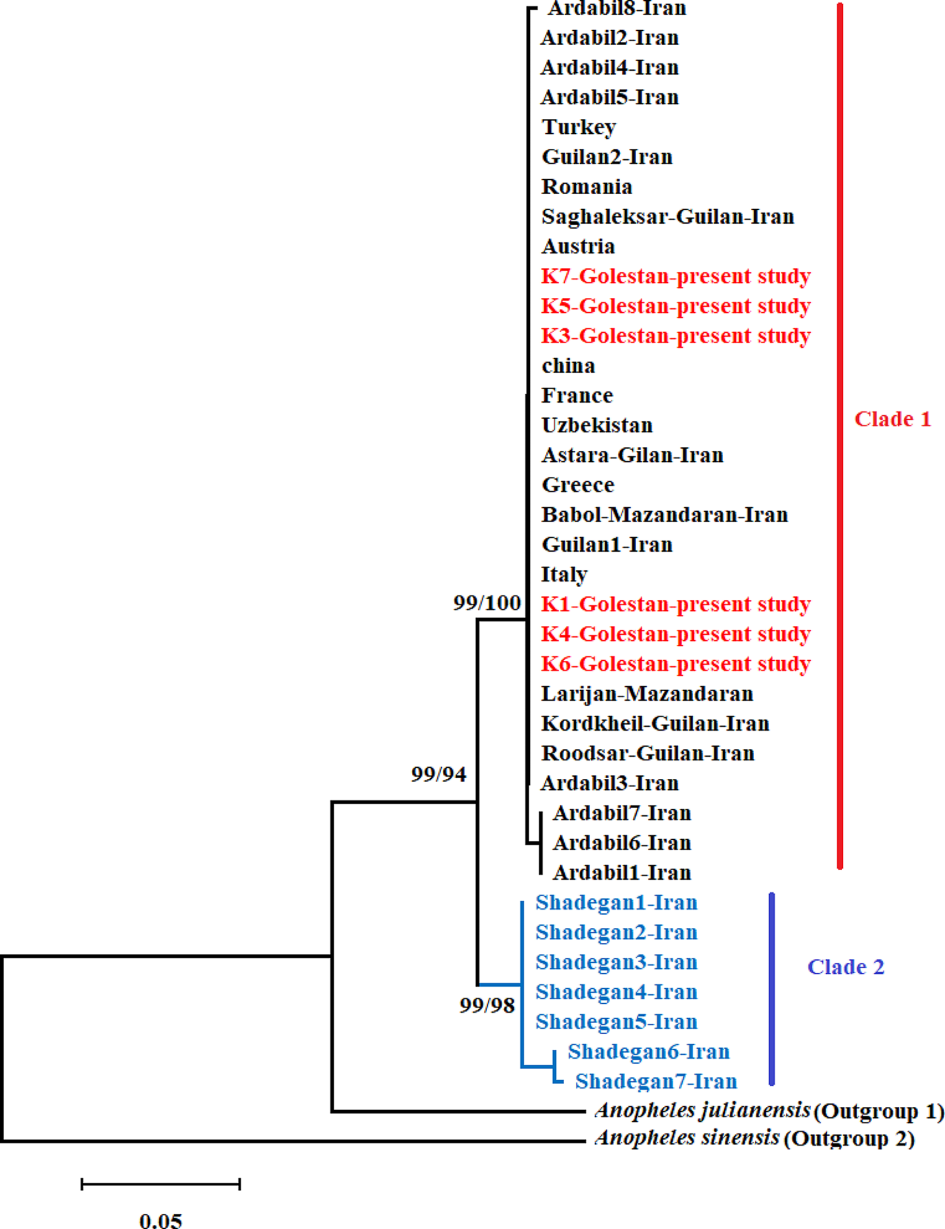
Evolutionary relationships of *Anopheles hyrcanus* based on the ITS2 gene sequences from the present study and GenBank (NCBI), inferred using the Neighbor-Joining (NJ), and Maximum likelihood (ML) method. Bootstrap values for NJ (10,000 replicates) before slash and for ML (1000 replicate) after slash are shown above or below the branches. *Anopheles julianensis* and *An. sinensis* were used as the outgroup

All phylogenetic analyses based on the ML, BI, and NJ methods (Figs. 2 and 3), revealed a monophyletic lineage for *An. hyrcanus* supported by high BI posterior probability and bootstrap values. Examining the similarity of the sequences of species of *Hyrcanus* Group from the GenBank sequence database (NCBI) and our original data based on both COI (Fig. 2) and ITS2 (Fig. 3) datasets showed that our sequences from northern Iran belonged to *An. hyrcanus*.

Based on the COΙ gene, overall, of the seven *An. hyrcanus* samples from Iran in the present study and the 12 sequences retrieved from the GenBank, the molecular analysis revealed 11 haplotypes, with a haplotype diversity (Hd) and haplotype diversity variance of 0.918 and 0.002, respectively. Among the 593 nucleotides examined, only one site was found to be non-synonymous, and the average number of nucleotide differences (k) was 4.550. Nucleotide diversity (Pi) of the *An. hyrcanus* sequences based on the COΙ gene was found to possess a low value of 0.0076, and the number of polymorphic sites (segregating) was 16.

Two highly supported major clades with bootstraps more than of 94 were recovered from NJ and ML analysis (Fig. 3) based on the ITS2 gene sequences. Clade 1 comprised the individuals from the present study, Iran and other countries from Europe and Asia, while Clade 2 included the individuals from southwestern Iran (Shadegan, Khuzistan Province) (Fig. 3). Percentage of the Kimura 2-parameter (K2P) mean genetic distance between these two main clades in the *An. hyrcanus* was 3.6% (Table 3). The lowest K2P interspecies distance in the *Anopheles* genus was observed between *An. lesteri* and *An. paraliae* with the value of 8% (Table 3).

**Table 3:** Percentage of the Kimura 2-parameter (K2P) mean genetic distance between *Anopheles* species based on the ITS2 gene sequences

***1-An. hyrcanus (clade1)***	**1**	**2**	**3**	**4**	**5**	**6**	**7**	**8**	**9**	**10**	**11**	**12**	**13**	**14**	**15**
***2-An. hyrcanus (clade2)***	**3.6%**	*													
***3-An. sineroides***	**27.6%**	**28.8%**	*												
**4** ***-An. peditaeniatus***	**41.5%**	**42.4%**	**45.3%**	*											
**5** ***-An. paraliae***	**32.5%**	**32.7%**	**20.4%**	**51.1%**	*										
**6** ***-An. nitidus***	**46.2%**	**48.7%**	**44.5%**	**61.5%**	**54.8%**	*									
**7** ***-An. nigerrimus***	**48.5%**	**49.7%**	**51.4%**	**67.2%**	**59.6%**	**22.8%**	*								
**8** ***-An. pursati***	**46.7%**	**49.8%**	**53.5%**	**63.3%**	**60.2%**	**20.4%**	**24.4%**	*							
**9** ***-An. belenrae***	**34.8%**	**35.0%**	**25.0%**	**47.9%**	**33.6%**	**56.8%**	**61.5%**	**65.1%**	*						
**10** ***-An. pullus***	**12.2%**	**12.8%**	**29.8%**	**41.5%**	**33.5%**	**48.0%**	**50.7%**	**49.9%**	**37.5%**	*					
**11** ***-An. liangshanensis***	**24.1%**	**25.1%**	**9.5%**	**44.0%**	**22.2%**	**49.4%**	**51.1%**	**52.5%**	**25.3%**	**24.5%**	*				
**12** ***-An. kweiyangensis***	**27.7%**	**27.5%**	**9.8%**	**46.1%**	**21.0%**	**48.4%**	**53.4%**	**53.4%**	**26.1%**	**27.8%**	**7.8%**	*			
**13** ***-An. lesteri***	**39.3%**	**40.8%**	**25.2%**	**55.2%**	**8.0%**	**59.4%**	**67.5%**	**66.5%**	**38.7%**	**40.8%**	**27.5%**	**25.5%**	*		
**14** ***-An. yatsushiroensis***	**12.2%**	**12.8%**	**29.8%**	**41.5%**	**33.5%**	**48.0%**	**50.7%**	**49.9%**	**37.5%**	**0.0%**	**24.5%**	**27.8%**	**40.8%**	*	
**15** ***-An. sinensis***	**31.8%**	**32.0%**	**22.4%**	**46.3%**	**29.0%**	**56.4%**	**61.0%**	**62.4%**	**8.8%**	**32.7%**	**19.6%**	**21.2%**	**34.6%**	**32.7%**	*
**16** ***-An. engarensis***	**26.9%**	**28.2%**	**18.4%**	**43.3%**	**25.9%**	**50.5%**	**57.6%**	**56.0%**	**14.7%**	**29.0%**	**19.6%**	**19.6%**	**31.0%**	**29.0%**	**11.9%**

**Fig. 4:**
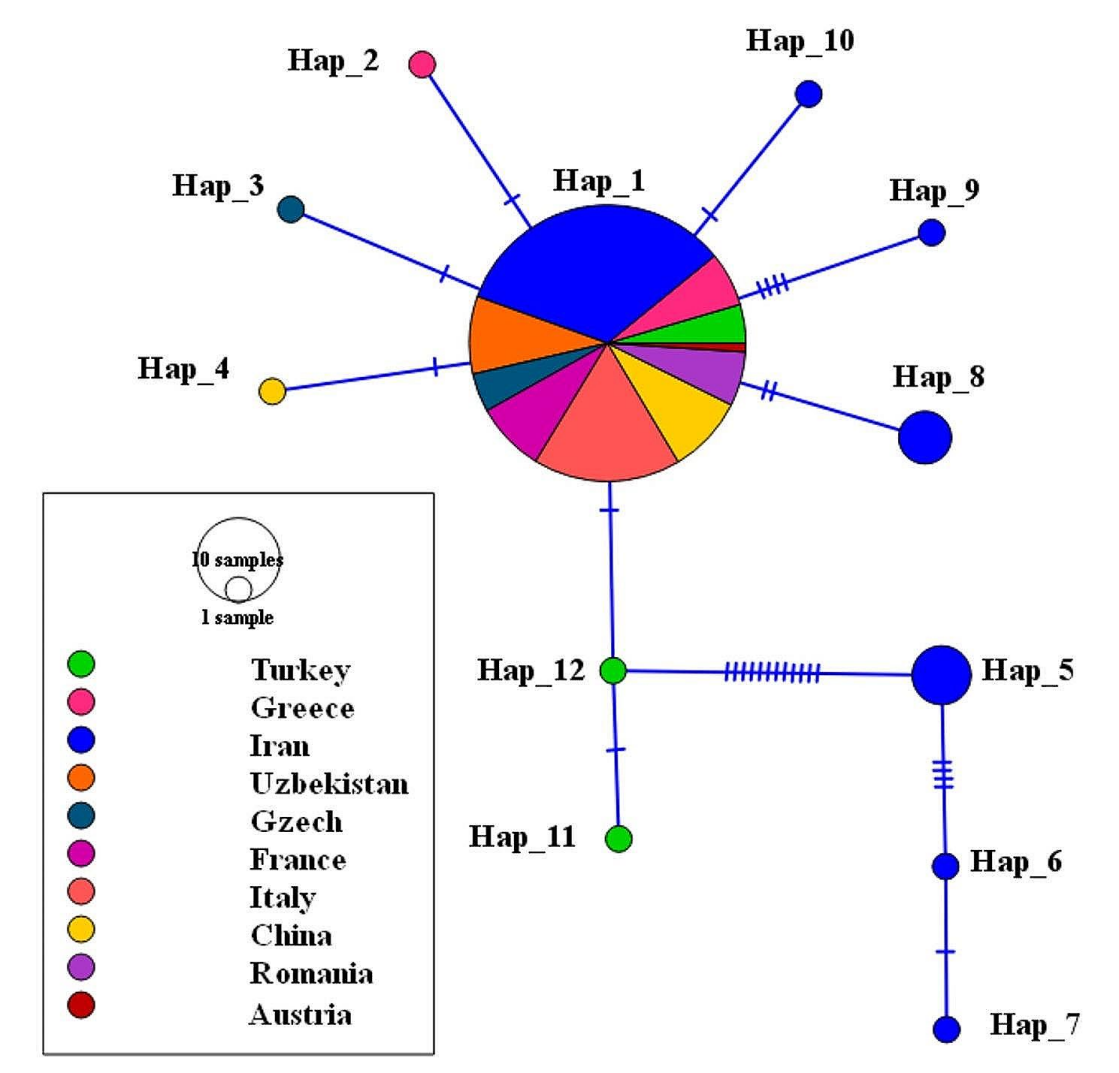
Median-joining haplotype network obtained for 487 bp of 128 nuclear ITS2 sequences of *An. hyrcanus* from the present study and the GenBank (NCBI). Circle size is relative to haplotype frequency; Hatch marks on the line represent mutational steps between haplotypes. Haplotype colors represent geographic locations of haplotypes as indicated on the right corner of the figure

Based on the 456 bp (excluding sites with gaps / missing data) of the ITS2 gene examined in all 128 sequences, 28 sites were polymorphic including nine singleton variable sites (two variants) and 19 parsimony informative sites (18 sites with two variants and one with three variants), resulting in the identification of 12 haplotypes (Fig. 4). In total, nucleotide diversity (Pi), and haplotype diversity (Hd) were 0.0039 and 0.262 respectively.

The haplotype network of ITS2 sequences recovered the existence of two main haplogroups distinct from each other by 12 mutational steps between Haplotype 12 from Turkey and Haplotype 5 from Iran (Fig. 4). One haplogroup included the haplotypes (Haplotype 5, 6, and 7) from southwestern Iran (Shadegan, Khuzistan Province) and, the second included all other haplotypes from Northern Iran and other countries from Europe and Central/Eastern Asia. These two Haplogroups were well corresponded to Clade 1 and 2 of *An. hyrcanus* in the phylogenetic tree (Fig. 3).

Of the 12 haplotypes, seven haplotypes were observed in Iran from which only one common haplotype was shared between Iran and other countries in Asia and Europe (Fig. 4). The haplotype 1 with a frequency of 110 was the most frequent and oldest haplotype which was shared between all countries in Asia and Europe. All samples in the present study have also belonged to this old haplotype. There were 6 distinct and new haplotypes from Iran, two from Turkey, one from each of the countries China, and one from Gzech (Fig. 4). The highest values of haplotype diversity and nucleotide diversity were seen in the sequences from Turkey, and Iran respectively (Table 4).

**Table 4:** Summary statistics of molecular diversity within ITS2 sequences of *an. hyrcanus*; total number of sites (excluding sites with gaps / missing data) were 456. N – number of Individuals, h – number of haplotypes, S- number of polymorphic (segregating) sites, K- average number of nucleotide differences, pi – nucleotide diversity, hd – haplotype diversity, Hap-Haplotype. The overall number of 128 includes the sequences from this study and all individuals from GenBank.

	Turkey		Greece	Iran	Uzbekistan	Gzech	France	Italy	China	Romania	Austria	Total Data Estimates
***n***	**7**		**8**	**50**	**10**	**6**	**9**	**19**	**11**	**7**	**1**	**128**
**h**	**3**		**2**	**7**	**1**	**2**	**1**	**1**	**2**	**1**	**1**	**12**
**S**	**2**		**1**	**24**	**0**	**1**	**0**	**0**	**1**	**0**	**0**	**28**
**K**	**0.76**		**0.25**	**4.04**	**0**	**0.333**	**0**	**0**	**0.18**	**0**	**0**	**1.8**
**Pi**	**0.0016**		**0.0005**	**0.009**	**0**	**0.0007**	**0**	**0**	**0.0004**	**0**	**0**	**0.0039**
**Hd**	**0.52**		**0.25**	**0.44**	**0**	**0.333**	**0**	**0**	**0.18**	**0**	**0**	**0.262**
**Hap1**	**5**		**7**	**37**	**10**	**5**	**9**	**19**	**10**	**7**	**1**	**110**
**Hap2**	**0**		**1**	**0**	**0**	**0**	**0**	**0**	**0**	**0**	**0**	**1**
**Hap3**	**0**		**0**	**0**	**0**	**1**	**0**	**0**	**0**	**0**	**0**	**1**
**Hap4**	**0**		**0**	**0**	**0**	**0**	**0**	**0**	**1**	**0**	**0**	**1**
**Hap5**	**0**		**0**	**5**	**0**	**0**	**0**	**0**	**0**	**0**	**0**	**5**
**Hap6**	**0**		**0**	**1**	**0**	**0**	**0**	**0**	**0**	**0**	**0**	**1**
**Hap7**	**0**		**0**	**1**	**0**	**0**	**0**	**0**	**0**	**0**	**0**	**1**
**Hap8**	**0**		**0**	**4**	**0**	**0**	**0**	**0**	**0**	**0**	**0**	**4**
**Hap9**	**0**		**0**	**1**	**0**	**0**	**0**	**0**	**0**	**0**	**0**	**1**
**Hap10**	**0**		**0**	**1**	**0**	**0**	**0**	**0**	**0**	**0**	**0**	**1**
**Hap11**	**1**		**0**	**0**	**0**	**0**	**0**	**0**	**0**	**0**	**0**	**1**
**Hap12**	**1**		**0**	**0**	**0**	**0**	**0**	**0**	**0**	**0**	**0**	**1**

## Discussion

The *An. hyrcanus* group comprises a minimum of 25 species and is categorized within the Myzorhynchus series of Anopheles, with one provisional designated member [23, 24]. These species are widely distributed across the Oriental and Palaearctic regions including Iran and encompass several species capable of transmitting some major vector-borne diseases including malaria, Japanese encephalitis virus, and filariasis [23].

According to morphological characteristics, the Hyrcanus group can be divided into three subgroups: the Nigerrimus subgroup, comprising of *An. nigerrimus*, *An. nitidus*, *An. pursati*, and *An. pseudosinensis*; the Lesteri subgroup, consisting of *An. lesteri*, *An. paraliae*, *An. peditaeniatus*, *An. crawfordi*, and *An. vietnamensis*; and species within an unassigned subgroup [23].

Due to the significant morphological similarity, certain species within the Hyrcanus group pose taxonomic controversies. Consequently, molecular techniques have emerged as a crucial foundation for the precise identification of these closely related species. The mitochondrial cytochrome c oxidase subunit region (COI) and the internal transcribed spacer 2 (ITS2) have been frequently utilized to tackle taxonomic issues within the Hyrcanus group owing to its low intraspecific and high interspecific variability, as indicated in numerous studies [12, 23]. These standard barcodes are effectively utilized for species identification and assessing interspecific hybridization. Utilizing data from the GenBank database and their original research dataset, the researchers employed 461 ITS2 sequences from 19 species and 466 COI sequences from 18 species to reconstruct the molecular phylogeny of the Hyrcanus group spanning its global geographic distribution [23, 25, 26].

The findings of the molecular analyses within this research clearly establish that all specimens gathered in the northeastern region of Iran are attributed to the *An. hyrcanus* species. The results derived from the phylogenetic and haplotype network studies unequivocally highlight the formation of a unique clade by the samples originating from northern Iran, in addition to those from various distribution regions of this species across Asia and Europe. Conversely, samples procured from the southwestern part of Iran are shown to constitute a separate and distinct clade based on the evidence presented (Fig. 3).

The observed genetic diversity between the aforementioned clades is quantified at 3.6%, as detailed in Table 1. It is worth highlighting that specimens derived from the southwestern region of Iran showcase a unique haplogroup. This distinct genetic profile, showcasing significant deviation from samples of identical species across various regions in Asia and Europe, suggests the emergence of a discrete population at the subspecies level. However, additional molecular studies focusing on different genes are increasingly recommended to validate this matter.

This occurrence can be ascribed to a multitude of factors. Among the foremost influencers is the significant climatic and geographical heterogeneity present in Iran, a pivotal element contributing to the proliferation of biodiversity within the nation. Geographically, Iran encompasses two distinct Palearctic regions situated in the northern and central territories, alongside the Oriental region located in the southern expanse [27]. The stark contrasts in weather patterns and vegetation across these regions offer a plausible rationale for the outcomes gleaned from this study. Furthermore, the escalating pace of climate change in recent times may exacerbate the geographical isolation of species, potentially leading to the emergence of novel subspecies and species within the area.

The considerable diversity in haplotypes and nucleotides identified within samples originating from Iran and Turkey emphasizes the importance of the geographical positioning and ecological variety present in this specific region of species dispersion. This genetic diversity could potentially have a vital impact on the emergence of populations that demonstrate resistance against a range of insecticides and display unique behaviors among vectors.

A similar study was conducted by Dinparast Djadid et al. on the *An. hyrcanus* Group in three Iranian provinces confirmed the findings of this study. They utilized only the ITS2 gene for molecular identification. Sequencing led to the discovery of a new member of the Hyrcanus group (referred to as *An. hyrcanus* spIR, a world record) in Iran, alongside *An. hyrcanus* Pallas. Consistent with our results, their phylogenetic analysis based on ITS2 indicated that Iranian Hyrcanus populations were clustering into two branches. Nevertheless, they demonstrated the evolutionary relatedness among Western and Eastern Palearctic taxa within the Hyrcanus Group [15].

## Conclusions

*An. hyrcanus* from southwestern Iran displayed a distinct haplogroup, suggesting that due to its genetic divergence from other specimens of the same species in various regions of Iran and other countries, it likely constitutes a separate population at the subspecies level. Certainly, further detailed and comprehensive supplementary studies are necessary to validate this hypothesis. Because a considerable number of the primary vectors pertain to the Hyrcanus Group, it is of paramount importance to undertake precise species identification and phylogenetic relationship evaluation within this group. This will play a crucial role in comprehending the transmission of malaria and other significant mosquito-borne illnesses.

Considering the challenges associated with solely relying on morphological characteristics for identifying cryptic species, there is a clear necessity for a thorough molecular phylogenetic survey on a significant scale across all provinces of Iran. This approach is crucial in gaining a more accurate understanding of the population genetic structure of this significant vector.

## Data Availability

The datasets generated and/or analysed during the current study are available in the GenBank™ repository, [Accession numbers: PP422139, PP422140, PP422141, PP422142, PP422143, PP422144, and PP422145].

## Abbreviations

ITS2: Internal transcribed spacer 2
COI: Cytochrome c oxidase I
COII: Cytochrome oxidase subunit II
An: Anopheles
UV: Ultraviolet
DNA: Deoxyribonucleic acid
PCR: Polymerase chain reaction
AIC: Akaike information criteria
BIC: Bayesian information criteria
ML: Maximum Likelihood
PopART: Population Analysis with Reticulate Trees
BLAST: Basic local alignment search tool
NCBI: National center for biotechnology information
Hd: Haplotype diversity
K2P: Kimura 2-parameter
Bp: Base pair
